# Obesity and Musculoskeletal Health of Young and Older Malaysian Women: A Cross-Sectional Study

**DOI:** 10.21315/mjms2023.30.1.12

**Published:** 2023-02-28

**Authors:** Nurdiana Zainol Abidin

**Affiliations:** 1 Department of Community Health, Advanced Medical and Dental Institute, Universiti Sains Malaysia, Pulau Pinang, Malaysia; 2 School of Biosciences, Faculty of Science and Engineering, University of Nottingham Malaysia, Selangor, Malaysia

**Keywords:** obesity, sarcopenia, osteoporosis, elderly, physical performance, menopause

## Abstract

**Background:**

Asian women are more susceptible to musculoskeletal disorders compared to their Caucasian counterparts, and employed women are substantially more prone to musculoskeletal disorders compared to men. Data on musculoskeletal health in Malaysian women are lacking. The study’s goal was to evaluate the body composition and functional performance of older and younger Malaysian women for obesity and musculoskeletal health problems.

**Methods:**

The study included 141 post-menopausal Malaysian women and 118 young Malaysian women between 18 years old and 32 years old of age. Body composition, bone density, handgrip strength and physical performance were assessed using bio-electrical impedance analyser, calcaneal quantitative ultrasound, hand dynamometer and modified short physical performance battery test, respectively.

**Results:**

There was a higher prevalence of ‘low muscle mass’ among the younger age group compared to their older counterparts (48 young women [40.0%] versus 44 post-menopausal women [31.2%]). Conversely, there was a higher prevalence of ‘obesity’ and ‘low bone density’ among the older age group compared to their younger counterparts. Mean broadband ultrasound attenuation (BUA) for both age groups was ≥ 70.0 dB/MHz. The majority of post-menopausal women had a ‘minor functional decline’ (40.6%), followed by moderate (28.1%), major (22.7%), severe (6.3%) and the lowest percentage for ‘no decline’ (2.3%).

**Conclusion:**

There was a high prevalence of obesity with poor musculoskeletal health in older Malaysian women, which may lead to frailty and higher incidences of falls and fractures at an advanced age. The screening of musculoskeletal conditions among Malaysian women may aid in early detection of abnormalities and timely intervention.

## Introduction

Musculoskeletal disorders, which comprised of more than 150 types, are known to affect both the elderly and young adults. While musculoskeletal problems are more common in older people, they are also found in young people, especially during their peak earning years (1). Musculoskeletal health is essential for living a healthy, active, and productive life. Therefore, it is critical to identify and implement effective interventions for people with musculoskeletal disorders (2).

The risk of musculoskeletal disorders significantly increases once a woman reaches menopause. Reduced oestrogen levels have been known to cause muscle and bone wasting, leading to musculoskeletal disorders such as sarcopenia and osteoporosis (3). These disorders significantly affect the locomotor system (i.e. muscles, bones and joints), leading to reduced mobility, dexterity, and the inability to maintain economic, social and functional independence. The disorders get worse when combined with obesity, which is expected to rise dramatically in developing countries (4, 5). In 2015, the World Health Organization (WHO) commissioned a paper that detailed the impact of musculoskeletal health issues on aging (6). Reduced physical capability (grip strength, walking speed, chair rising times and standing balance times) is linked to persistent pain, poor mobility and function, lower quality of life and mental well-being (7). Frailty is usually one of the main clinical outcomes of these disorders.

The aim of this study was to assess the musculoskeletal health of postmenopausal Malaysian women and to compare it with women of the younger age group (18 years old–32 years old). The screening of musculoskeletal health among Malaysian women allows for early detection of abnormalities and timely intervention. Furthermore, Malaysia lacks data on musculoskeletal values of young women, making it difficult to determine cut-points to diagnose disorders such as sarcopenia and osteoporosis. This study aims to bridge this gap in knowledge by describing the mean values for muscle mass, bone density, and physical performance of young and older Malaysian women.

## Methods

### Selection and Recruitment of Participants

A total of 141 post-menopausal Malaysian women (aged 45 years old–88 years old) were recruited from various locations around the Semenyih and Klang Valley areas of Kuala Lumpur, Malaysia. One hundred and eighteen (*n* = 118) young women (aged 18 years old–32 years old) were recruited from the University of Nottingham Malaysia during term time (October 2017–April 2018) and were undergraduate students and staff members. No menstrual period, bleeding or spotting for 12 months before enrolment were considered post-menopausal. Details about the study, including the aims, procedures, advantages, risks and potential discomforts, were briefed to the participants prior to enrolment. The following inclusion criteria were used for eligibility: older cohort: i) woman; ii) citizen of Malaysia (of Malay, Indian or Chinese ethnicity) and iii) postmenopausal (no menstrual period, bleeding or spotting for 12 months before enrolment); younger cohort: i) woman ii) citizen of Malaysia (of Malay, Indian or Chinese ethnicity) and iii) aged 18 years old–32 years old. The exclusion criteria included: i) inability to stand for height, weight and gait speed assessments; ii) presence of artificial limbs and/or metal implants; iii) severe cardiac, pulmonary or musculoskeletal disorders; iv) severe cognitive impairment or any disability that prevents communication and v) terminal illness.

### Anthropometric and Body Composition Measurements

#### Height

A portable stadiometer was used to measure height to the nearest 0.1 cm (SECA 217, Vogel & Halke GmbH & Co., Germany). Participants were instructed to stand with their shoulders, buttocks and heels against the stadiometer, with their toe tips creating a 45° angle, heels touching, head held straight and neck in a natural position.

#### Body Fat

A segmental bio-electrical impedance analyser was used to determine body fat (InBody 230 Body Composition Analyzer, Biospace Co. Ltd., Korea). The participant’s weight was automatically calculated while on this equipment.

#### Waist Circumference

The waist circumference (WC) was measured with a measuring tape (SECA 203, GmbH & Co. Kg., Hamburg, Germany). With patients standing erect, waist circumference (cm) was measured at the midpoint between the final rib and the anterior superior iliac spine.

#### Quantitative Ultrasound (QUS) Bone Assessments

A calcaneal ultrasound bone densitometer (SAHARA^®^ Clinical Bone Sonometer, Hologic Inc., Waltham, MA, USA) was used to determine bone density. The broadband ultrasonic attenuation (BUA) (dB/MHz), which represents attenuated sound waves, and the speed of sound (SOS) (m/s), were measured simultaneously using the SAHARA^®^ system. A comparison measurement was done through a reference medium to evaluate the sound attenuation of the heel alone, without any bias resulting from the transducers and/or transducer pads. The SAHARA® QC Phantom (included with the SAHARA^®^ device) served as the reference medium.

#### Appendicular Skeletal Muscle Mass Index (appSMMI)

A segmental bio-electrical impedance analyser was used to determine body composition (BIA, InBody 230 Body Composition Analyzer, Biospace Co. Ltd., Seoul, Korea). The total of the muscle masses of the four limbs was used to compute appendicular skeletal muscle mass. The total of the muscle masses of the four limbs, adjusted for height in squared metres (kg/height^2^), was defined as the appendicular skeletal muscle mass index (appSMMI).

#### Muscle Strength

Handgrip strength was evaluated in each hand using a hand dynamometer (JAMAR Hydraulic Hand Dynamometer® Model PC-5030 J1, Fred Sammons, Inc., Burr Ridge, IL: USA) as a proxy for muscular strength. For each hand, handgrip strength was assessed twice, with the highest of the two values considered in the analysis. The American Society of Hand Therapists recommends the following positioning: participant seated, shoulders adducted and neutrally rotated, elbow flexed at 90°, forearm in neutral, and wrist between 0° and 30° of dorsiflexion (8).

### Functional Performance

#### Short Physical Performance Battery Test

The modified short physical performance battery (SPPB) test was used to assess functional performance. The following tests were performed under the SPPB based on the recommendations of Ilich et al. (9): one-leg stance (to evaluate balance), gait speed (to measure endurance) and the sit-to-stand chair test (to assess lower extremity strength). The cut-off value for each test was ≤ 16 s, < 0.8 m/s and ≤ 20 times, respectively. The SPPB has a 0.76 internal consistency and predictive validity for mortality, nursing home admission and disability risk (10).

#### One-Leg Stance

For one-leg stance, measurements were taken for both the right and left legs. The contestants stood on one leg for a maximum of 30 s while elevating the other limb. When the participant contacts any surface or lowers the other limb to the ground or at the conclusion of 30 s, the test comes to an end (9).

#### Gait Speed

A 6-metre normal walk was timed to determine gait speed. Two cones or pieces of tape were used to mark the 6-metre course, which was measured with a roll-up, self-retracting construction measuring tape. The participant was asked to walk at a normal pace from one end of the course to the other for the duration of the test. Timing began when the tester/instructor said ‘begin’ and ended when one of the participant’s feet crossed the 6-metre marker completely. Participants were allowed to use their canes or other walking aids during the test if they typically did so. Based on the Asian Working Group for Sarcopenia guidelines, the cut-off value is 0.8 m/s (11).

#### Sit-to-Stand Chair Test

Before the start of the test, the participant was instructed to sit in an armless chair with arms crossed over the chest, back straight and feet flat on the floor. The test demands the participant to rise from the chair and sit down as many times as possible for a maximum of 30 s. The number of successive chair sit-to-stand tests completed was recorded, with the final count being the last time the participant sat in the chair. A ‘fit’ participant was classified as someone who accomplished > 20 sit-to-stands in a 30-s interval and had good lower extremity strength (9, 12, 13).

### Statistical Analysis

The statistical tool SPSS was used to conduct the analysis (version 24 for Windows; SPSS, Inc., Chicago, IL, USA). The study participants’ characteristics were reported as mean and standard deviations or as the number of participants and the proportional representation. Frequency and percentages were reported for categorical variables. Differences between proportions were determined using Pearson’s chi-square test. A comparison between the mean parameter and standard cut-off was performed using one-sample *t*-test. A comparison of the distributions of various parameters between two groups was performed using independent *t*-test. Two-tailed *P*-value of ≤ 0.05 was recognised as statistically significant.

## Results

One hundred and forty-one postmenopausal women (aged 45 years old–88 years old) and 118 young Malaysian women (aged 18 years old–32 years old) were included in the analysis. The ethnic distribution of the participants was 36.0% Malays (*n* = 51), 30.0% Chinese (*n* = 42) and 34.0% Indians (*n* = 48) for post-menopausal women, and 25.0% Malays (*n* = 30), 48.0% Chinese (*n* = 56) and 27.0% Indians (*n* = 32) for young women.

Categorisation based on body mass index (BMI) shows that the prevalence of ‘obesity’ (BMI ≥ 27.5 kg/m^2^) was higher among postmenopausal women compared to their younger counterparts (48.0%, *n* = 68 versus 14.0%, *n* = 17, respectively). A similar pattern was also seen in the ‘overweight’ (BMI = 23 kg/m^2^–27.49 kg/m^2^) category (28.0%, *n* = 40 versus 19.0%, *n* = 23, respectively). Conversely, the percentage for ‘normal’ (BMI = 18.5 kg/m^2^–22.99 kg/m^2^) was higher in young women compared to their older counterparts (49.0%, *n* = 58 versus 21.0%, *n* = 29, respectively) (Tables 1 and 2). There was also a higher prevalence of ‘underweight’ (BMI < 18.5 kg/m^2^) among the young women compared to the older age group (18.0%, *n* = 21 versus 2.8%, *n* = 4, respectively). Alternatively, if categorisation was based on WC and body fat percent (BFP), the prevalence of obese/overweight category was also higher among the older age group (WC = 63.0% versus 18.0%, and BFP = 86.0%, versus 48.0%) (Tables 1 and 2).

Additionally, the mean for all three markers for obesity (BMI, WC and BFP) in postmenopausal women was significantly higher than their respective standard cut-off values (*P* < 0.01) (Table 1). Conversely, in young women, a significant difference was only found in WC, which was lower than the standard cut-off value (71.9 [9.3] cm < 80.0 cm [standard cut-off]), *P* < 0.001) (Table 2).

Appendicular skeletal muscle mass index (appSMMI) for both age groups was found to be significantly higher than the standard cutoff values (*P* < 0.001), indicating a good level of muscle mass (Tables 1 and 2). Interestingly, there was a higher prevalence of ‘low muscle mass’ in the younger age group compared to their older counterparts (young women = 40.0% versus post-menopausal women = 31.2%) (Tables 1 and 2).

Based on the results for BUA (dB/MHz), it was revealed that most participants in both age groups had a healthy level of bone density (≥ 70.0 dB/MHz). Only 18.0% (*n* = 25) of postmenopausal women and < 1.0% of younger women were categorised as ‘osteopenic/osteoporotic’ based on the standard cut-off (< 54 dB/MHz) (Tables 1 and 2).

The background data such as education level, smoking habit, alcohol-drinking habit, physical activity level and co-morbidities were gathered only from post-menopausal women. The majority of the participants (77.0%) had completed secondary education and were non-smokers (98.6%), non-drinkers (97.8%) and non-milk drinkers (70.4%). Approximately, half of our patients (50.4%) said that they were ‘inactive’ during the week, while the other half said they did at least 10 min of physical activity per day (other than normal types of activity like housework). Forty-three percent of participants said they had never been diagnosed with any of the diseases identified in the survey (Table 1).

Table 3 shows the scores for the functional performance of the participants. Overall scores show that most of the participants had ‘minor functional decline’ (40.6%), followed by moderate (28.1%), major (22.7%), severe (6.3%) and the lowest percentage for ‘no decline’ (2.3%).

When divided by ethnicity, this study found that most Malays and Chinese had ‘minor functional decline’ (53% and 55%, respectively), followed by ‘moderate decline’, ‘major decline’, ‘severe decline’ and finally, ‘no decline’. However, the same pattern was not seen for Indians. The majority of Indians were in the ‘major decline’ category (43.0%), followed by ‘moderate decline’ (37.1%), ‘severe decline’ (14.3%) and ‘minor decline’ (5.7%). There were no participants in the ‘no decline’ category among the Indians.

Table 4 shows a comparison of characteristics between young and postmenopausal women. To limit biases, postmenopausal women with a diagnosis of musculoskeletal-related disorders were excluded from the analysis (i.e. osteoarthritis, rheumatoid arthritis, osteoporosis and people who had previously suffered a stroke). Ultimately, data from 118 healthy post-menopausal women were analysed and compared to that from young women (*n* = 118).

In comparison to their older counterparts, younger women were significantly taller with stronger handgrip strength and denser bone mass, while the older counterparts were heavier (body weight and BMI) with a larger midsection (WC) and had a higher BFP (*P* ≤ 0.05). Their muscle mass indices (FFMI, SMMI and appSMMI) were similarly substantially greater (*P* ≤ 0.05) than those of their younger peers.

## Discussion

In this study, the musculoskeletal health of young and older women was compared. Southeast Asians generally have a lower muscle mass profile in relation to stature and total body mass compared to their European counterparts (14). Low muscle mass is proposed as one of the primary reasons of developing non-communicable diseases (NCDs) such as type-2 diabetes mellitus (T2DM) at lower BMIs in Southeast Asian populations as compared to others (15). Obesity and lifestyle factors such as diet and sedentary lifestyle play important roles in the development of NCD, the variations between populations are also related to the syndrome. Tillin et al. (16) found that Southeast Asians living in London, UK, had twice the risk of developing T2DM, with onset typically occurring 5 years earlier and at a lower BMI (by 5 kg/m^2^) compared to those of European ancestry (16) owing to the low lean mass in this population (15). Therefore, it is important to determine the cause of such variations in musculoskeletal health at any given time. In this study, we investigated age and ethnic disparities among Malaysian women in relation to bone density, muscle mass, fat mass and functional performance to develop a musculoskeletal health profile of the population.

In our cohort, the post-menopausal women were found to be moderately educated (having completed secondary school or higher), non-smokers, non-drinkers of alcohol and non-drinkers of milk (Table 1). Interestingly, half of the cohort (50.4%) reported being ‘inactive’ during the week, while another half reported having some physical activity (other than regular types of activity such as household chores) for at least 10 min per day, as reflected by their BMIs. Categorisation based on BMI showed that the prevalence of obesity and overweight in postmenopausal women was 48.0% and 28.0%, respectively. Although the physical activity level of the young adult group is not known, the body composition revealed a lower prevalence of obesity and overweight in the younger age group (14.0% and 19.0%, respectively) (Table 2). It shows that obesity rates are higher among the older population compared to those of their younger counterparts, implying an upward trend of adiposity with age. Even after using alternative adiposity indicators to determine obesity status (WC and BFP), the trend persists. In addition to overall adiposity, abdominal adiposity was also found to be lower in the younger group (Table 2) compared to that of their older counterparts (Table 1). Moreover, there was a higher percentage of normal-weight participants in the younger age group (49.0% [Table 2] versus 21.0% [Table 1]).

Although the National Health and Morbidity Survey Malaysia (NHMS) in 2011 and 2015 reported reduced increment rate of obesity among Malaysian adults, the prevalence of obesity in Malaysia is still among the highest in Southeast Asia (17). The latest NHMS study in 2019 reported that one in two adults in Malaysia were overweight (BMI > 25 kg/m^2^) or obese (BMI > 30 kg/m^2^). Among the age group of 55 years old–59 years old, 60.9% were found to be overweight or obese, and among the females aged ≥ 18 years old, 54.7% were found to be overweight or obese. Those of Indian ethnicity were found to have the highest percentage of overweight or obese people at 63.9% (18). In 2012, a study from Malaysia using BMI as the obesity indicator, showed that among 125 postmenopausal women aged 50 years old–65 years old, 80.0% were overweight and obese (19), which is consistent with our findings (76.0% in 2019). This means that for > 5 years, the rate has remained the same. In the case of the younger population, although the prevalence of overweight and obese people is lower, findings from our study showed an increase in the prevalence compared to that reported previously. Previous studies (2010–2011) on Malaysian undergraduates (aged 18 years old–25 years old) found that the prevalence of overweight among female students ranged from 6.11% (20) to 8.5% (21) and the prevalence of obesity ranged from 0.56% (20) to 3.8% (21). Evidently, these percentages were much lower than the findings from our study (19.0% of overweight and 14.0% of obesity), indicating a need for intervention. Interestingly, a Malaysian study published in 2019 also reported similar, and higher, overweight and obesity prevalence among Malaysian undergraduate students (23% and 17.6% of overweight and obesity, respectively) (22). Stress is a predominant factor associated with obesity and overweight in university students (23–25). Therefore, stress management intervention should be implemented among university undergraduates.

Apart from general obesity, information on the area of the body where fat tends to accumulate is also important, as some diseases tend to be significantly correlated with fat distribution. A review by Després (26) showed that a high WC was predictive of an increased risk of dyslipidaemia, hypertension, cardiovascular diseases and T2DM, independent of the BMI (26). WHO guidelines also state that alternative measures that reflect abdominal obesity, such as WC, are superior to overall BMI (27). Based on empirical research, WC was also found to be a better measurement of obesity compared to BMI (28). Additionally, WC was found to be a simpler and more accurate predictor of T2DM than other indices such as BMI and waist-to-hip (WHR) (29).

In this study, WC data showed that fat distribution differs between older and younger women. According to the WHO/International Association for the Study of Obesity/International Obesity Task Force (WHO/IASO/IOTF) (30) and the International Diabetes Federation (IDF) (31), a WC ≥ 80 cm for an Asian female was classified as abdominal obesity. It is well known that abdominal obesity increases with age. In our cohort, older and younger women had significantly higher and lower WC, respectively, as compared to the standard cut-off (Tables 1 and 2) (32–35). Although abdominal obesity in the general population has been increasing for several years (17.4% in 2006, 20.9% in 2011, 20.0% in 2014, 23.0% in 2015 and 50% in 2019) (18, 36), the prevalence of abdominal obesity is still lower in the younger population compared to older adults. The latest report in 2019 revealed that one in two Malaysian adults had abdominal obesity, where most of them were in the 60 years old–64 years old of age group (71.5%), of Indian ethnicity (68.3%) and females (64.8%) (18).

Overall, data for anthropometrics, body composition and bone density showed a high disparity between the younger and older age groups (Table 4). The most peculiar finding is the muscle mass profile of the two age groups. In this study, the younger age group was found to have significantly lower muscle mass compared to the older age group, which contradicts the current understanding of age-related loss of muscle mass (37). However, due to their young age, the low muscle mass is not necessarily indicative of ‘sarcopenia’, but rather inadequacy of mass due to low protein diet and sedentary lifestyle. Although sarcopenia is age-related, studies have shown that the correlation is not seen before 40 years old of age (38–41). Moreover, the level of muscle mass is related to stature. Therefore, the level of muscle mass may be higher in the older age group due to the higher levels of adiposity and overall body mass. Regardless, quantity does not equal quality. Although the younger age group had lower muscle mass, the group still performed better in handgrip strength compared to the older age group (Table 4).

In our cohort, bone density was determined by BUA (dB/MHz). The younger age group displayed significantly higher BUA compared to their older counterparts (Table 2 and Table 1, respectively). Nevertheless, most of the participants in both age groups had a BUA > 54 dB/MHz (42). This indicates a healthy bone density level. Conversely, only 18.0% of postmenopausal women had BUA < 54 dB/MHz (42) and only one person in the younger age group was categorised as such. According to Johansen et al. (42) who measured bone mineral density (BMD) at the lumbar spine and hip of 73 women aged 29 years old–86 years old (mean 65 years old) using dual x-ray absorptiometry (DXA), people with BUA < 54 dB/MHz threshold value tend to have low femoral neck density. In the absence of DXA, QUS is an adequate substitute that provides wider public accessibility as it is portable, user-friendly, cost-effective and does not emit ionising radiation (43).

### Functional Performance of Multi-Ethnic Post-menopausal Malaysian Women

In 2016, Ilich et al. (9) proposed the measurements of handgrip strength and three modified components of SPPB to assess the functional performance of obese postmenopausal women with musculoskeletal disorder (osteosarcopenic obesity). The three tests were gait speed, one-leg stance,- and a sit-to-stand test. Based on these criteria and scoring references, the current study assessed the functional performance of the older age group (Table 3a). Table 3b outlines the assessment criteria and the corresponding scores. Overall, most of the current study’s population had minor functional decline at 40.6%, while ‘no decline’ was only 2.3% (Table 3), indicating an adequate overall physical performance profile. When stratified by ethnicity, the Malays and Chinese presented similar patterns, with most of the groups having only minor functional decline (53% and 55%, respectively) (Table 3). The Indians were found to have the highest percentage in the ‘major decline’ category (43.0%), followed by moderate decline (37.1%), a severe decline (14.3%) and a minor decline (5.7%). There were no participants in the ‘no decline’ category among the Indians (Table 3). Previous studies have reported similar findings. A study on musculoskeletal pain among Malaysian multi-ethnic groups found that Indian women had the highest musculoskeletal pain rate (28.4%) compared to Malay and Chinese (44). Moreover, this ethnic group also had the highest number of people with fibromyalgia. Similar findings were also found among rural women in Bhigwan, Western India (45). Veerapen et al. (44) theorised that the shared risk factors responsible for this may be cultural and genetic. The high propensity for older Indian women (especially those living in developing countries) to report musculoskeletal pain may also be related to numerous household tasks, poor posture and ergonomics, as well as psychosocial stress (44).

## Conclusion

To conclude, there was a higher prevalence of obesity in combination with poor musculoskeletal health in older Malaysian women, compared to their younger counterparts, which may lead to frailty and a higher incidence of falls and fractures at advanced age. Therefore, the screening of musculoskeletal conditions among Malaysian women should be conducted in a wider population to ensure early detection of abnormalities and timely intervention. Furthermore, suboptimal muscle mass was found to be more prevalent in the younger age group compared to their older counterparts. For most women, muscle mass begins to decline after the age of 30. Aging causes anabolic impairments in skeletal muscle, which leads to reductions in muscle mass and strength, both of which are directly related to mortality rates in the elderly. Therefore, it is important to accumulate an optimal peak muscle mass before it starts to decline. A wider campaign is required to increase the awareness of accumulating a healthy amount of muscle mass at young age and the importance of its preservation in old age.

### Limitations and Strengths

This study has some limitations. First, the older participants were mostly healthy and relatively young (~60 years old), resulting in a group with generally good functional performance, limiting the generalisability of the results. Second, the SPPB test was not assessed among the younger group, prohibiting us from determining the differences in functional performance between the two age groups. Finally, even though we used statistical inference techniques, biases may have existed due to the fact that the participants were mostly volunteers. These participants could be more health-conscious and be willing to undertake a 1-h interview, including an SPPB test and body composition assessments, than a random sample of the population.

One of this study’s strengths is the diversity of the sample, which comprised Malaysia’s three major ethnic groups including Malay, Indian or Chinese, ensuring its validity. Furthermore, the older cohort were not institutionalised, allowing for easy extrapolation to all older people. In addition, we studied the musculoskeletal health of both younger and older Malaysian women. To the best of our knowledge, this is the first study to investigate age disparities in musculoskeletal statuses between younger and older Malaysian women, thereby bridging the research gap.

## Figures and Tables

**Table 1:** Characteristics and body composition measurements of post-menopausal women, *N* = 141 (Malay, *n* = 51; Chinese, *n* = 42; Indian, *n* = 48)

Variables		*N*	Mean (SD)	Minimum-maximum	Normal range and standard cut-off	*P*-value	Mean difference	95% CI of difference
Age (years old)		141	60.4 (7.5)	45.0–88.0	na			
Age at menarche (years old)		125	13.3 (1.5)	9.0–17.0	na			
Age at menopause β (years old)		121	50.5 (4.1)	36.0–59.0	na			
Years since menopause		121	9.5 (7.3)	1.0–35.0	na			
Height (cm)		141	153.1 (6.2)	137.5–169.0	na			
Weight (kg)		141	63.4 (12.6)	31.9–100.9	na			
BMI (kg/m^2^)	All	141	27.1 (5.3)	15.4–43.0	23.0	**< 0.001** *	+4.1	3.2, 4.9
	Normal	29	20.8 (1.3)	18.5–22.8	18.5–22.99ƚ			
	Overweight	40	25.2 (1.4)	23.1–27.4	23.0–27.49ƚ			
	Obese Type 1 and 2	68	31.4 (3.7)	27.5–43.0	≥ 27.5ƚ			
WC (cm)	All	139	84.2 (12.6)	55.1–121.0	80.0	**< 0.001** *	+4.2	2.1, 6.3
	Overweight/Obese	87	91.6 (9.3)	80.0–121.0	≥ 80.0δ			
Body fat (%)	All	141	40.5 (7.8)	20.7–54.0	32.0	**< 0.001** *	+8.5	7.2, 9.8
	Obese	121	42.8 (5.7)	32.3–54.0	≥ 32.0γ			
FFMI (kg/m^2^)		141	15.8 (1.7)	11.8–21.8				
SMMI (kg/m^2^)		141	8.4 (1.1)	6.0–12.8				
AppSMMI (kg/m^2^)	All	140	6.1 (0.9)	4.0–10.7	5.7	**< 0.001** *	+0.43	0.3, 0.6
	Sarcopenic	44	5.2 (0.4)	4.01–5.69	≤ 5.7ƒ			
BUA (dB/MHz)	All	139	70.0 (16.8)	35.9–122.2	54.0	**< 0.001** *	+15.9	13.1, 18.7
	Osteopenic	25	47.5 (5.3)	35.9–53.8	< 54.0μ			

Variables	*N*			*n*	Percentage (%)
Highest education level	140	No formal education		10	7.1
		Primary school		22	15.7
		Secondary school		55	39.3
		Certificate/Diploma		29	20.7
		University degree		19	13.6
		Postgraduate degree		5	3.6
Cigarette smoking status	139	Non-smoker		137	98.6
		Current smoker		2	1.4
Alcohol drinking	139	Non-drinker		136	97.8
		Current drinker		2	2.2
Milk drinking	135	Non-drinker		95	70.4
		Drinker	1 serving/day	33	24.4
			2 or more servings/day	7	5.2
Self-rated PA status	137				
		Inactive		69	50.4
		Active (at least 10 min per day)		68	49.6
Disease(s)/disorder(s)	139				
		None		60	43.2
		Hypertension		53	
		T2DM		31	
		Heart problems		11	
		Osteoarthritis		12	
		Rheumatoid Arthritis		7	
		Osteoporosis		8	
		Have had stroke		4	
		Depression/anxiety		6	

Notes: All participants reported no menstrual bleeding or spotting for at least 1 year prior to enrolment;

β Only 121 participants remembered the exact age they reached menopause;

BMI = body mass index; four participants (*n* = 4) were underweight BMI < 18.5 kg/m^2^; FFMI = fat free mass index; SMMI = skeletal muscle mass index; SD = standard deviation; CI = confidence interval;

* analysed using one-sample *t*-test, (overall mean versus respective cut-points;

BMI = 23 kg/m^2^; WC = 80 cm; BFP = 32%; appendicular SMMI = 5.7 kg/m^2^; *P* < 0.001);

γ Ilich et al. (9);

ƒ AWGS (Asian Working Group for Sarcopenia) (11);

δ WHO (27);

ƚ Asian BMI cut points by WHO expert consultation (46);

μ Johansen et al. (42)

**Table 2:** Characteristics and body composition measurements of young women, *N* = 118 (Malay, *n* = 30; Chinese, *n* = 56; Indian, *n* = 32)

Variables		*n*	Mean (SD)	Minimum-maximum	Normal range and standard cut-off	*P*-value	Mean difference	95% CI of difference
Age (years old)	118		22.1 (2.2)	18.0–32.0	na			
Height (cm)	118		159.3 (5.5)	142.6–173.0	na			
Weight (kg)	118		57.0 (11.6)	39.0–100.8	na			
BMI (kg/m^2^)	All	118	22.4 (4.5)	16.3–40.3	23.0	0.346	−0.40	−1.3, 0.4
	Normal	58	20.5 (1.4)	18.5–22.8	18.5–22.99ƚ			
	Overweight	23	25.1 (1.2)	23.1–27.2	23.0–27.49ƚ			
	Obese Types 1 and 2	17	31.3 (3.7)	27.5–40.3	≥27.5ƚ			
WC (cm)	All	106	71.9 (9.3)	59.0–99.5	80.0	**<0.001** *	−7.70	−9.5, −5.9
	Overweight/Obese	19	88.1 (6.2)	80.0–99.5	≥80.0δ			
Body fat (%)	All	118	32.4 (7.7)	17.4–53.3	32.0	0.312	+0.73	−0.7, 2.2
	Obese	57	39.2 (5.9)	32.1–53.3	≥32.0γ			
FFMI (kg/m^2^)	118		14.8 (1.5)	10.8–18.8				
SMMI (kg/m^2^)	118		8.0 (0.9)	5.5–10.3				
AppSMMI (kg/m^2^)	All	118	5.9 (0.7)	4.1–7.6	5.7	**<0.001** *	+0.25	0.1, 0.4
	Low muscle mass	48	5.2 (0.3)	4.01–5.69	≤5.7ƒ			
BUA (dB/MHz)	All	118	86.5 (16.3)	52.7–132.2	54.0	**<0.001** *	+32.5	29.6, 35.3
	Low bone mass	1	52.7	–	<54.0μ			

Notes: BMI = body mass index; twenty participants (*n* = 20) were underweight BMI < 18.5 kg/m^2^; FFMI = fat free mass index; SMMI = skeletal muscle mass index; SD = standard deviation; CI = confidence interval;

* analysed using one-sample *t*-test, (overall mean versus respective cut-points;

BMI = 23 kg/m^2^, WC = 80 cm, BFP = 32%, appendicular SMMI = 5.7 kg/m^2^, *P* < 0.001),

γ Ilich et al. (9);

ƒ AWGS (Asian Working Group for Sarcopenia) (11);

δ WHO (27);

ƚ Asian BMI cut points by WHO expert consultation, (46);

μ Johansen et al. (42)

**Table 3a:** Assessment and scoring of the functional performance of post-menopausal women

Functional performance/Ethnicity	Severe functional decline (total score = 0)		Major functional decline (total score = 1)		Moderate functional decline (total score = 2)		Minor functional decline (total score = 3)		No decline (total score = 4)		*P*-value
				
	*n*	%	*n*	%	*n*	%	*n*	%	*n*	%	
Overall (*n* = 128)	8	6.3	29	22.7	36	28.1	52	40.6	3	2.3	< 0.001†*
Malay (*n* = 51)	2	3.9	9	17.6	12	23.5	27	52.9	1	2.0	
Chinese (*n* = 42)	1	2.4	5	11.9	11	26.2	23	54.8	2	4.8	
Indian (*n* = 35)	5	14.3	15	42.9	13	37.1	2	5.7	0	0.0	

Notes:

† analysed using Pearson chi-square test (ethnicities * functional performance categories);

* *P* < 0.001

**Table 3b:** Scoring reference

Functional status	Handgrip strength† (< 18 kg)	One-leg stance‡ (≤ 16 s)	Gait speed† (< 0.8 m/s)	Sit-to-stand chair test‡ (≤ 20 times)	Total score
Severe functional decline	0	0	0	0	0
Major functional decline*	0	1	0	0	1
Moderate functional decline**	0	0	1	1	2
Minor functional decline***	0	1	1	1	3

No functional decline 1 1 1 1 4 Notes: The score of ‘0’ is assigned to each test performed at or below the given cut-off and the score of ‘1’ to each test performed above the cut-off value;

* Any one performance could be scored as ‘1’ if it is above the cut-off for a given functionality;

** Any two performances could be scored as ‘1’ if they are above the cut-off for given functionality;

*** Any three performances could be scored as ‘1’ if they are above the cut-off for given functionality; A total score of 0 indicates a state of severe functional decline. A total score of 1 indicates a state of major functional decline. A total score of 2 indicates moderate functional decline. A total score of 3 indicates minor functional decline. A total score of 4 indicates no functional decline;

† Criteria proposed by the Asian Working Group for Sarcopenia (AWGS) (11);

‡ Ilich et al. (9)

**Table 4:** Differences in characteristics: young versus post-menopausal women

Variables	*n*	Young women Mean (SD)	*n*	Post-menopausal women Mean (SD)	*P-value	Cohen’s *d* (Effect size)
Age (years old)	118	22.1 (2.2)	118	60.0 (7.8)	**< 0.001**	−6.574
Height (cm)	118	159.3 (5.5)	118	152.8 (6.2)	**< 0.001**	1.104
Weight (kg)	118	56.9 (11.6)	118	63.9 (12.6)	**< 0.001**	−0.586
BMI (kg/m^2^)	118	22.4 (4.5)	118	27.4 (5.3)	**< 0.001**	−1.015
WC (cm)	106	71.9 (9.3)	115	84.8 (12.5)	**< 0.001**	−1.171
BFP (%)	118	32.4 (7.7)	118	41.1 (7.6)	**< 0.001**	−1.133
FFMI (kg/m^2^)	118	14.8 (1.5)	118	15.8 (1.7)	**< 0.001**	−0.599
SMMI (kg/m^2^)	118	8.0 (0.9)	118	8.5 (1.1)	**< 0.001**	−0.482
AppSMMI (kg/m^2^)	118	5.9 (0.7)	118	6.1 (0.9)	**0.05**	−0.248
HGS (kg)	117	24.7 (4.2)	112	19.8 (5.0)	**< 0.001**	1.058
BUA (dB/MHz)	117	86.5 (16.3)	116	70.3 (17.2)	**< 0.001**	0.970
SOS (m/s)	117	1570.9 (33.1)	116	1525.0 (31.9)	**< 0.001**	1.415
Est. BMD (g/m^2^)	117	0.610 (0.122)	116	0.449 (0.124)	**< 0.001**	1.310
QUI/Stiffness	117	108.1 (19.9)	116	82.3 (21.0)	**< 0.001**	1.268
T-score (MY Ref.)	117	−0.4 (1.1)	116	−1.3 (1.0)	**< 0.001**	1.323
Z-score	117	−0.1 (1.0)	116	0.0 (1.0)	0.34	−0.131

Notes: Post-menopausal women with musculoskeletal-related disorders were excluded from analysis (i.e osteoarthritis, rheumatoid arthritis, osteoporosis and people who had previously suffered stroke); SD = standard deviation; CI = Confidence interval; BMI = body mass index; WC = waist circumference; BFP = body fat percent; FFMI = fat free mass index; SMMI = skeletal muscle mass index; AppSMMI = appendicular skeletal muscle mass index; HGS = hand grip strength; BUA = broadband ultrasonic attenuation; Est. BMD = estimated bone mineral density; SOS = speed of sound; QUI = quantitative ultrasonic index, MY = Malaysia;

* analysed using independent *t*-test; formula for Cohen’s *d* = t √(N1+N2/N1*N2), small = 0.2, medium = 0.5, large = 0.8

## References

[1] Briggs AM, Woolf AD, Dreinhöfer K, Homb N, Hoy DG, Kopansky-Giles D (2018). Reducing the global burden of musculoskeletal conditions. Bull World Health Organ.

[2] Oakman J, Keegel T, Kinsman N, Briggs AM (2016). Persistent musculoskeletal pain and productive employment; a systematic review of interventions. Occup Environ Med.

[3] Khadilkar SS (2019). Musculoskeletal Disorders and Menopause. J Obstet Gynaecol India.

[4] Kelly T, Yang W, Chen CS, Reynolds K, He J (2008). Global burden of obesity in 2005 and projections to 2030. Int J Obes (Lond).

[5] Deparment of Social and Preventive Medicine Obesity in Malaysia is a ticking time bomb [Internet].

[6] World Health Organization (WHO) (2015). World report on ageing and health.

[7] Cooper R, Kuh D, Hardy R, Mortality Review Group, FALCon HALCyon Study Teams (2010). Objectively measured physical capability levels and mortality: systematic review and meta-analysis. BMJ.

[8] Fess E (1992). Clinical assessment recommendations.

[9] Ilich JZ, Kelly OJ, Inglis JE (2016). Osteosarcopenic obesity syndrome: what is it and how can it be identified and diagnosed?. Curr Gerontol Geriatr Res.

[10] Guralnik JM, Simonsick EM, Ferrucci L, Glynn RJ, Berkman LF, Blazer DG (1994). A short physical performance battery assessing lower extremity function: association with self-reported disability and prediction of mortality and nursing home admission. J Gerontol.

[11] Chen LK, Liu LK, Woo J, Assantachai P, Auyeung TW, Bahyah KS (2014). Sarcopenia in Asia: consensus report of the Asian Working Group for Sarcopenia. J Am Med Dir Assoc.

[12] Shin H, Liu PY, Panton LB, Ilich JZ (2014). Physical performance in relation to body composition and bone mineral density in healthy, overweight, and obese postmenopausal women. J Geriatr Phys Ther.

[13] Jones CJ, Rikli RE, Beam WC (1999). A 30-s chair-stand test as a measure of lower body strength in community-residing older adults. Res Q Exerc Sport.

[14] Rush EC, Freitas I, Plank LD (2009). Body size, body composition and fat distribution: comparative analysis of European, Maori, Pacific Island and Asian Indian adults. Br J Nutr.

[15] Pomeroy E, Mushrif-Tripathy V, Cole TJ, Wells JCK, Stock JT (2019). Ancient origins of low lean mass among South Asians and implications for modern type 2 diabetes susceptibility. Sci Rep.

[16] Tillin T, Sattar N, Godsland IF, Hughes AD, Chaturvedi N, Forouhi NG (2015). Ethnicity-specific obesity cut-points in the development of Type 2 diabetes – a prospective study including three ethnic groups in the United Kingdom. Diabet Med.

[17] Chan YY, Lim KK, Lim KH, Teh CH, Kee CC, Cheong SM (2017). Physical activity and overweight/obesity among Malaysian adults: findings from the 2015 National Health and morbidity survey (NHMS). BMC Public Health.

[18] Institute for Public Health 2020 National Health and Morbidity Survey (NHMS) 2019: non-communicable diseases, healthcare demand, and health literacy—Key findings.

[19] Hasnah H, Amin I, Suzana S (2012). Bone health status and lipid profile among post-menopausal malay women in Cheras, Kuala Lumpur. Malays J Nutr.

[20] Huda N, Ahmad R (2010). Preliminary survey on nutritional status among university students at Malaysia. Pakistan J Nutri.

[21] Gan WY, Mohd NM, Zalilah MS, Hazizi AS (2011). Differences in eating behaviours, dietary intake and body weight status between male and female Malaysian University students. Malays J Nutr.

[22] Wan Mohamed Radzi CWJ, Salarzadeh Jenatabadi H, Alanzi ARA, Mokhtar MI, Mamat MZ, Abdullah NA (2019). Analysis of obesity among Malaysian university students: a combination study with the application of Bayesian structural equation modelling and pearson correlation. Int J Environ Res Public Health.

[23] Odlaug BL, Lust K, Wimmelmann CL, Chamberlain SR, Mortensen EL, Derbyshire K (2015). Prevalence and correlates of being overweight or obese in college. Psychiatry Res.

[24] Gupta S, Ray TG, Saha I (2009). Overweight, obesity and influence of stress on body weight among undergraduate medical students. Indian J Community Med.

[25] Kim J-I (2016). Predictors of weight control behavior according to college students’ BMI, perception of body shape, obesity stress, and self-esteem. J Korea Academia-Industrial Cooperation Soc.

[26] Després JP (2012). Body fat distribution and risk of cardiovascular disease: an update. Circulation.

[27] World Health Organization (WHO) (2011). https://apps.who.int/iris/handle/10665/44583.

[28] Yang F, Lv JH, Lei SF, Chen XD, Liu MY, Jian WX (2006). Receiver-operating characteristic analyses of body mass index, waist circumference and waist-to-hip ratio for obesity: screening in young adults in central south of China. Clin Nutr.

[29] Mamtani MR, Kulkarni HR (2005). Predictive performance of anthropometric indexes of central obesity for the risk of type 2 diabetes. Arch Med Res.

[30] World Health Organization (WHO) (2000). Regional Office for the Western, P. The Asia-Pacific perspective: redefining obesity and its treatment.

[31] International Diabetes Federation (2006). The IDF consensus worldwide definition of the metabolic syndrome.

[32] López-Sobaler AM, Rodríguez-Rodríguez E, Aranceta-Bartrina J, Gil Á, González-Gross M, Serra-Majem L (2016). General and abdominal obesity is related to physical activity, smoking and sleeping behaviours and mediated by the educational level: findings from the ANIBES study in Spain. PLoS ONE.

[33] Kowalkowska J, Poínhos R, Franchini B, Afonso C, Correia F, Pinhão S (2016). General and abdominal adiposity in a representative sample of Portuguese adults: dependency of measures and socio-demographic factors’ influence. Br J Nutr.

[34] Krzysztoszek J, Wierzejska E, Zielińska A (2015). Obesity: an analysis of epidemiological and prognostic research. Arch Med Sci.

[35] Wang H, Wang J, Liu MM, Wang D, Liu YQ, Zhao Y (2012). Epidemiology of general obesity, abdominal obesity and related risk factors in urban adults from 33 communities of Northeast China: the CHPSNE study. BMC Public Health.

[36] Institute for Public Health (2015). National Health and Morbidity Survey 2015 (NHMS 2015). II. Non-communicable diseases, risk factors and other health problems.

[37] Zhu Kun, Kerr Deborah A, Meng Xingqiong, Devine Amanda, Solah Vicky, Binns Colin W (2015). Two-year whey protein supplementation did not enhance muscle mass and physical function in well-nourished healthy older postmenopausal women. J Nutr.

[38] Baier S, Johannsen D, Abumrad N, Rathmacher JA, Nissen S, Flakoll P (2009). Year-long changes in protein metabolism in elderly men and women supplemented with a nutrition cocktail of beta-hydroxy-beta-methylbutyrate (HMB), L-arginine, and L-lysine. JPEN J Parenter Enteral Nutr.

[39] Flakoll P, Sharp R, Baier S, Levenhagen D, Carr C, Nissen S (2004). Effect of beta-hydroxy-beta-methylbutyrate, arginine, and lysine supplementation on strength, functionality, body composition, and protein metabolism in elderly women. Nutrition.

[40] Grimby G, Danneskiold-Samsøe B, Hvid K, Saltin B (1982). Morphology and enzymatic capacity in arm and leg muscles in 78–81 years old men and women. Acta Physiol Scand.

[41] Janssen I, Heymsfield SB, Wang ZM, Ross R (2000). Skeletal muscle mass and distribution in 468 men and women aged 18–88 year. J Appl Physiol.

[42] Johansen A, Evans W, Stone M (1999). Bone assessment in elderly women: what does a low bone ultrasound result tell us about bone mineral density?. Arch Gerontol Geriatr.

[43] Laugier P (2004). An overview of bone sonometry. International Congress Series.

[44] Veerapen K, Wigley RD, Valkenburg H (2007). Musculoskeletal pain in Malaysia: a COPCORD survey. J Rheumatol.

[45] Chopra A, Saluja M, Patil J, Tandale HS (2002). Pain and disability, perceptions and beliefs of a rural Indian population: a WHO-ILAR COPCORD study. WHO-International League of Associations for Rheumatology. Community oriented program for control of rheumatic diseases. J Rheumatol.

[46] World Health Organization Expert Consultation (2004). Appropriate body-mass index for Asian populations and its implications for policy and intervention strategies. Lancet.

